# Cost analysis of multiple sclerosis in Brazil: a cross-sectional multicenter study

**DOI:** 10.1186/s12913-016-1352-3

**Published:** 2016-03-24

**Authors:** Nilceia Lopes da Silva, Maira L. S. Takemoto, Alfredo Damasceno, Yara D. Fragoso, Alessandro Finkelsztejn, Jefferson Becker, Marcus V. M. Gonçalves, Charles Tilbery, Enedina M. L. de Oliveira, Dagoberto Callegaro, Fernanda C. Boulos

**Affiliations:** Novartis Biociências SA, Av. Professor Vicente Rao, 90. 04636-000, São Paulo, SP Brazil; ANOVA Tradução do Conhecimento em Saúde, Rio de Janeiro, RJ Brazil; Medical School, Universidade Estadual de Campinas, Campinas, SP Brazil; Medical School, Universidade Metropolitana de Santos, Santos, SP Brazil; Department of Neurology, HC de Porto Alegre, Porto Alegre, RS Brazil; Hospital São Lucas da PUCRS, Porto Alegre, RS Brazil; Hospital Regional Hans Dieter Schimidt, Joinville, SC Brazil; Division of Neurology, Santa Casa de Misericórdia de São Paulo, São Paulo, SP Brazil; Universidade Federal de São Paulo, São Paulo, Brazil; Hospital das Clínicas da USP, São Paulo, SP Brazil

**Keywords:** Multiple sclerosis, Cost analysis, Cost of illness, Quality of life

## Abstract

**Background:**

Multiple sclerosis (MS) is a central nervous system disease associated with irreversible progression of disability, which imposes a substantial socioeconomic onus. The objective of this study was to determine the economic impact of multiple sclerosis from the Brazilian household and healthcare system perspectives. Secondary objectives were to assess the impact of fatigue on daily living and health-related quality of life (HRQL) of MS patients.

**Methods:**

This is a cross-sectional study in which Brazilian eligible patients attending eight major MS specialized sites answered an interview capturing data on demographics, disease characteristics and severity, comorbidities, resource utilization, fatigue, utilities and health-related quality of life from November/2011 to May/2012 . Costs were assessed considering a prevalence-based approach within 1 year of resource consumption and were estimated by multiplying the amount used by the corresponding unit cost. Patients were classified as having mild, moderate or severe disability according to the Expanded Disability Status Scale (EDSS).

**Results:**

In total, 210 patients who met eligibility criteria were included, 40 % had mild, 43 % moderate and 16 % severe disability; disability level was missing for 1 %. The average total direct cost per year was USD 19,012.32 (SD = 10,465.96), and no statistically significant differences were not observed according to MS disability level (*p* = 0.398). The use of disease modifying therapies (DMTs) corresponded to the majority of direct expenditures, especially among those patients with lower levels of disability, representing around 90 % of total costs for mild and moderate MS patients. It was also observed that expenses with medical (except DMTs) and non-medical resources are higher among patients with more severe disease. Worsening disability also had an important influence on health-related quality of life and self-perceived impact of fatigue on daily living.

**Conclusion:**

Our data demonstrates the significant economic impact of MS on both Brazilian household and health system, in terms of DMTs and other disease management costs. When patients move upwards on the disease severity scale, costs with health resources other than drugs are significantly increased.

## Background

Multiple sclerosis (MS) is an inflammatory and demyelinating disease of the central nervous system that can cause several symptoms that include upper and lower extremity disabilities, visual disturbances, balance and coordination problems, spasticity, altered sensation, abnormal speech, swallowing disorders, fatigue, bladder and bowel problems, sexual dysfunction, and cognitive and emotional disturbances [1–3]. It is estimated that about 2.5 million people are living with the disease worldwide [4] and in Brazil, MS prevalence is estimated to range from 1.36 to 20/100,000 inhabitants, depending on the characteristics of the studied population [4, 5]. Although people can be diagnosed at any age, MS is usually diagnosed during early adulthood and the average age of MS onset is 30 years [4]. MS can be found in four different forms (relapsing-remitting, primary progressive, progressive relapsing and secondary progressive). The relapsing-remitting type is the most frequent at the time of diagnosis (about 85 %) and it is estimated that up to 80 % of these patients will evolve to the secondary progressive form [4].

Despite its low prevalence relative to other chronic diseases, such as diabetes and coronary artery diseases, MS imposes a considerable socioeconomic onus [6, 7]. Analyses of MS costs conducted in several countries have demonstrated that costs are largely driven by the progression of patients to severe stages of disability [8, 9]. Compared to mild disability, severe disability is associated with increased costs of hospitalizations, consultations, laboratory tests and other drugs, although the cost of immunomodulatory drugs is low [8, 9]. Indeed, direct medical costs in the early disease stages are outweighed by indirect costs, associated with decreased work and productivity, in later stages [10]. Furthermore, intangible non-monetary adverse impacts of MS, such as health-related loss of quality of life and symptoms like fatigue, represent an important concern for patients and families [11–13]. Within the scope of economic analysis in health care, patients’ quality of life is usually translated into a measure related to economic theory named utility which was also previously described for MS [3, 8, 9, 14–18].

So far, there are few studies that addressed costs associated with MS in Brazil. All studies focused on medical direct costs, especially those related to the use of disease modifying therapies (DMTs), and analyses were conducted from the Brazilian Unified Health System (SUS) perspective [19–23]. In fact, DMTs are available to MS patients in the public sector through the Specialized Component of Pharmaceutical Assistance Program of SUS [24–26].

In the Brazilian health system, the public (SUS) and private sectors (Supplementary Health System and out-of-pocket) are distinct but interconnected, and people can use services provided by either sectors depending on ease of access or their ability to pay [27]. Currently, SUS is a universal system in which the government, at federal, state and city levels, provides health services funded by taxes [27]. Besides SUS, around 25 % of Brazilians, mostly employees from public and private companies, are also covered by the Supplementary Health System (SH) constituted by different private health insurance plans [27]. SUS and SH have specific lists of coverage including medications, exams, consultations and hospitalizations [28–30]. In the out-of-pocket subsector, services are directly paid by patients within the private health market [27].

In the current scenario of increasing healthcare costs, with limited budget and higher demand for new technologies, cost-of-illness studies can be relevant to support policy-makers in the resource allocation process [31, 32]. Considering this, it is important to quantify the costs of MS to better understand the impact of the disease not only on the health system, but also on patients and their families.

Although there are some reports describing MS-related costs funded by the public health sector [19–23], there is a lack of studies describing MS-related costs including healthcare system and Brazilian household perspectives, i.e., considering direct costs funded by SUS, SH and directly paid by patients and their families. Thus, the primary aim of the study was to assess the economic impact of MS from the Brazilian household and healthcare system perspectives. Secondary objectives were to assess the impact of fatigue on daily living and health-related quality of life (HRQL) of MS patients.

## Methods

### Study design and patient assessment

This was a multicenter, cross-sectional study conducted in eight sites, 5 public, 1 private and 2 with mixed funding, specialized in MS treatment in Southern and Southeastern regions of Brazil. Patients were screened for eligibility and consecutively invited to participate, as they attended a routine visit at study sites. If they agreed, they were asked to sign an informed consent. Patients were deemed eligible if they were at least 18 years old and if they had clinical diagnosis of MS according to the revised McDonald criteria [33]. Patients were excluded if they had any physical or mental condition that would impair their understanding and ability to answer the study interview (particularly the self-reported measures of HRQL and fatigue) and/or if they were already enrolled in a clinical trial at the time of enrolment. This study was approved by the independent Ethics Committees of each participating site. During data collection, which occurred between November/2011 and May/2012, patients answered a face-to-face structured interview in Portuguese, conducted by a clinical research assistant during an outpatient routine visit. This interview aimed to collect self-reported variables about socio-demographics and clinical aspects, disability level, professional activities, including early retirement and sick leaves, HRQL and impact of fatigue on daily living (both measured using validated questionnaires as described in the sessions below) and health resource utilization.

### Disability Level

The Expanded Disability Status Scale (EDSS) was used to assess patients’ disability level. It is a self-reported instrument composed by 20 items with scores ranging from 0 to 10 points, increasing in accordance with the disability level. This is a well-established method used in clinical trials, epidemiological studies and in clinical practice [34]. Patients were classified as having mild, moderate or severe disability according to the following cutoffs: 0–3 (mild), 4–6.5 (moderate), and ≥7 (severe), as previously described by other authors [14, 35].

### Health-related quality of life and utility measures

Quality of life and utility measures are different concepts that are related to each other. Utility derives from Neumann and Morgenstern’s economic theory for the decision making process, in which a model was developed to represent how people make decisions when they face uncertainty conditions, based on individual preferences [36]. So, utility measures come from an individual judgment of different health statuses and summarize concepts related to health, well-being and quality of life. Utility measures are usually used to define public health policies, resource allocation and the evaluation of services and programs, as a proxy of how people value specific changes in health status related to new therapies, for example [36]. Measures usually range from 0 to 1, and are conceptually valued using as a reference death as the worst health state (equal to 0) and perfect health as the best health state (equal to 1).

Health-related Quality of life was assessed using the EQ-5D (EuroQol 5 Dimensions Questionnaire) which measures generic quality of life through questions assessing the level of impairment in five 5 different domains (mobility, self-care, usual activities, pain/discomfort and anxiety/depression) and an additional self-applicable visual analogue scale (EQ-VAS). Each domain has 3 response levels (no problem, some problem and serious problem) and in EQ-VAS patients graduate their general health status from 0 (worst imaginable health state) to 100 (best imaginable health state) [37].

Data obtained from EQ-5D were subsequently converted into a utility index, using the algorithm developed for the United Kingdom, as there were no conversion methods available for the Brazilian population at the time of development of the study protocol [38, 39].

### Impact of fatigue

The impact of fatigue on daily living of MS patients was assessed through the MFIS-BR (Modified Fatigue Impact Scale, Brazilian Portuguese version), which measures the impact of fatigue through 21 questions about physical, cognitive and psychosocial domains [40]. Impact of fatigue was considered as absent when total score was ≤38 points; low when the score was between 39 and 58 points; and high when ≥59 points [40].

### Productivity loss

Productivity loss was assessed through the analysis of early retirement due to MS and the occurrence of sick leaves for those currently working. These data were reported by patients during the face-to-face structured interview. Productivity-related variables were analyzed only to describe the overall impact of MS for patients, without converting them into monetary values.

### Resource utilization

To assess resource utilization, patients were asked by an interview, using a specific study questionnaire, about the consumption of specific MS-related health resources. Different recall periods were used for each type of resource: i) one month for assistance from family or friends; ii) three months for medications and assistance from contracted paid caregivers; iii) six months for outpatient visits and emergency department visits; iv) twelve months for relapses, hospitalizations, tests and major investments (home or vehicle adaptation, acquisition of walking aids, among others.). To ensure comparability, in a subsequent step all resources were annualized.

### Costs

Costs were assessed considering a prevalence-based approach and the time period of 1 year of resource consumption. Only costs directly related to diagnosis, management and treatment of MS were estimated. To better understand the funding of MS resources in the study, costs were divided according to the source of funding (the healthcare system or the patient/family), which defines different study economic perspectives – the perspective of the household (costs funded by patients and families, i.e., out-of-pocket expenses) and the perspective of the healthcare system (costs funded by SUS or SH). Direct costs related to MS management were estimated by multiplying the amount of resources used by the corresponding unit cost. Unit costs for each resource used (SH and/or SUS) were collected on July/2012 from the latest available value described in the Brazilian official price lists, in 2012 Brazilian Real values (BRL), as follows: Health Price Database from the Brazilian Ministry of Health, for medication [41]; Unified Health System Management System of the Table of Procedures, Medicines, Orthotics, Prosthetics and Special Materials (SIGTAP), for complementary exams, hospitalizations and consultations at SUS [42]; and Hierarchical Brazilian Classification of Medical Procedures (CBHPM) from the Brazilian Medical Association, for consultations in the private setting [43]. Expenses related to non-prescribed drugs (OTC medication), equipment and other investments and professional caregivers were reported by patients during the interview. These data were reported considering the source of funding, patients or healthcare system, and costs related to disease modifying therapies (DMTs) were reported separately, as patients with severe disability are not always treated with DMTs which can potentially be a confounding factor.

Costs were also analyzed according to the level of disability (EDSS), impact of fatigue (MFIS-BR cut-offs for impact of fatigue) and other patients’ demographics and clinical characteristics. Brazilian real values (BRL) were converted into American dollar values (USD) using the exchange rate of the date of consultation to the price list (08/24/2012), where USD1.00 corresponded to BRL 2.0255.

### Statistical analysis and sample size

Since a standard method for sample size calculation in cost of illness studies is not available and this is a part of a broad study that also assessed patients’ preferences related to MS treatment characteristics [44], sample size was calculated in order to evaluate that outcome. It was estimated that 210 respondents would be enough to obtain measures with a significance level of 5 %. For the HRQL outcome assessed through utility measures, the estimated sample size (N = 210) was validated, and using a SD = 0.32, the variability provided would be ≈ 0.08. Shapiro-Wilk and Jarque-Bera tests were used to test for normal distribution of data.

All data were submitted to exploratory analysis to describe measures of central tendency and dispersion for continuous variables and frequency for categorical variables. In the univariate analysis of total costs, comparisons between groups were performed using the chi-square of Pearson test (for frequencies) and ANOVA (normal distributions) with the post hoc Bonferroni or Kruskal-Wallis (non-normal distribution) test were used (for means). The same tests and criteria were used to compare means according to EDSS levels (mild, moderate and severe).

In the multivariate analysis, ANOVA with partial sums of squares (Partial SS) test was employed to analyze the association between MS-related direct costs and the following variables: EDSS level, gender, educational level, impact of fatigue level (MFIS scale), MS relapse, any self-reported comorbidities, type of MS, and occupation. All analyses were performed using the statistical software Stata (version MP11) and R Project (version 2.13.1). The 95 % confidence interval and a *p* value ≤0.05 were assumed for statistical significance.

### Ethics approval

A written consent was obtained from all the respondents before conducting the interviews. This study was approved by independent Ethics Committees of each participating research site: Comitê de Ética em Pesquisa da Universidade Metropolitana de Santos-UNIMES (no. 18/2011); Comitê de Ética em Pesquisa da Faculdade de Ciências Médicas da Universidade Estadual de Campinas - UNICAMP (no. 433/164 211); Comitê de Ética em Pesquisa da Irmandade da Santa Casa de Misericórdia de São Paulo - ISCMSP (no. 173/11); Comitê de Ética em Pesquisa do Hospital de Clínicas de Porto Alegre - HCPA (no. 166 110267); Comissão de Ética para Análise de Projetos de Pesquisa – CAPPesq da Diretoria Clínica do Hospital das Clínicas da Faculdade de Medicina da Universidade de São Paulo - FMUSP (no. 0571/11); Comitê de Ética em Pesquisa da Universidade Federal de São Paulo - UNIFESP/Hospital São Paulo (no. 0769/11); Comitê de Ética em Pesquisa da Pontifícia Universidade Católica do Rio Grande do Sul - PUCRS (no. 12/05750); Comitê de Ética em Pesquisa do Hospital Municipal São José – Joinville/SC (no. 12004).

## Results

### Patients’ demographics and clinical characteristics

In total, 210 patients who met eligibility criteria were included in the study. Most of them (70 %) were female, with a mean age of 40.7 (SD = 11.5) years old, living with family/spouse (94 %) and with an average monthly income of USD 806.47 (SD = 869.39). Patients’ EDSS classification was: 40 % had mild disability (EDSS 0–3), 43 % had moderate disability (EDSS 4–6.5), and 16 % had severe disability (EDSS 7–9). The majority of patients had relapsing-remitting disease (79 %) and 85 % of patients with severe disability (EDSS 7–9) had secondary progressive disease.

Occurrence of a relapse in the previous year was reported by 49 % of the total sample, being different across EDSS levels (Mild: 49 %; Moderate: 57 %; Severe: 24 %). Details on patients’ demographic and clinical characteristics are described in Table 1;a statistically significant difference among the severity groups was observed for age (*p* < 0.001), educational level (*p* = 0.044), living (*p* = 0.004), MS type (*p* < 0.001) and at least one episode of recurrence (*p* = 0.004).

**Table 1:** Patients’ demographics, labor force and clinical characteristics in each EDSS level

	Mild (EDSS 0–3) (N = 84)	Moderate (EDSS 4–6.5) (N = 91)	Severe (EDSS 7–9) (N = 33)	TOTAL (N = 210)^a^	*p*-values*
	N (%)	N (%)	N (%)	N (%)	
Age (years) (Mean [SD])	36.8 (10.1)	41.2 (11.4)	49.2 (10.9)	40.7 (11.5)	<0.001
Gender					0.056
Male	18 (21)	30 (33)	14 (42)	62 (29)	
Female	66 (79)	61 (67)	19 (58)	148 (70)	
Educational level					0.044
Never been to school	–	–	–	–	
Elementary school^b^	11 (13)	14 (16)	10 (30)	35 (16)	
High school^b^	34 (41)	53 (58)	13 (39)	100 (48)	
Higher education^b^	31 (37)	21 (23)	8 (24)	61 (29)	
Post-Graduation^b^	8 (10)	3 (3)	1 (3)	12 (6)	
No information	–	–	1 (3)	1 (1)	
Living					0.004
Alone	–	8 (9)	2 (6)	11 (5)	
Family/Spouse	84 (100)	83 (91)	30 (91)	198 (94)	
Home support or asylum	–	–	1 (3)	1 (1)	
Monthly income (USD$) (Mean [SD])	817.96 (899.18)	748.93 (790.96)	904.50 (994.75)	806.47 (869.39)	0.8925
MS type					<0.001
Relapse-remitting	84 (100)	75 (82)	5 (15)	166 (79)	
Secondary progressive	–	16 (18)	28 (85)	44 (21)	
Diagnosis time (years) (Mean [SD])	5.2 (4.4)	8.2 (5.8)	n.a.	7.9 (6.2)	
Comorbities	64 (76)	79 (87)	30 (91)	173 (82)	0.074
Relapses (Y/N)	41 (49)	52 (57)	8 (24)	102 (49)	0.004
Number of relapses in the last year (Mean [SD])	1.5 (1.0)	1.7 (1.0)	1.4 (0.5)	1.6 (1.0)	
Employed/Self-employed	51 (31)	17 (19)	0 (0)	70 (34)	<0.001
Sick leave due to Multiple Sclerosis^c^	15 (29)	11 (65)	n.a.	26 (37)	0.089
Sick leave duration (days) (Mean [SD])	170.5 (258.4)	90.9 (75.0)	n.a.	141.7 (198.7)	
Early retirement by disease or disablement	9 (11)	48 (53)	21 (64)	78 (37)	<0.001
Time between diagnosis and retirement (years) (Mean [SD])	4.9 (5.1)	3.5 (3.9)	4.2 (4.2)	3.9 (4.1)	
Age at retirement (years) (Mean [SD])	42 (12.0)	38 (8.9)	39 (9.9)	39 (9.5)	
Health-related Quality of Life (EQ-5D)					
Mobility					<0.001
no problem	56 (66.7)	7 (7.7)	–	64 (30)	
some problem	28 (33.3)	83 (91.2)	22 (66.7)	134 (64)	
serious problem	–	1 (1.1)	11 (33.3)	12 (6)	
Self-care					<0.001
no problem	77 (91.7)	51 (56.0)	4 (12.1)	133 (63)	
some problem	7 (8.3)	40 (44.0)	18 (54.5)	66 (31)	
serious problem	–	–	11 (33.3)	11 (5)	
Usual activities					<0.001
no problem	52 (61.9)	17 (18.7)	2 (6.1)	72 (34)	
some problem	31 (36.9)	68 (74.7)	15 (45.5)	115 (55)	
serious problem	1 (1.2)	6 (6.6)	16 (48.5)	23 (11)	
Pain/discomfort					0.001
no problem	39 (46.4)	21 (23.1)	7 (21.2)	68 (32)	
some problem	43 (51.2)	57 (62.6)	26 (78.8)	126 (60)	
serious problem	2 (2.4)	12 (13.2)	–	15 (7)	
no information	–	1 (1.1)	–	1 (1)	
Anxiety/depression					0.148
no problem	35 (41.7)	30 (33.0)	15 (45.5)	80 (38)	
some problem	42 (50.0)	45 (49.5)	17 (51.5)	106 (51)	
serious problem	7 (8.3)	16 (17.6)	1 (3.0)	24 (11)	
EQ-VAS (Mean [SD])	82.5 (13.7)	65.8 (18.5)	59.1 (17.5)	71.6 (18.9)	<0.001
Impact of fatigue on daily living (MFIS-BR)					<0.001
Absent	56 (67)	33 (36)	11 (33)	102 (49)	
Present - Low	17 (20)	34 (37)	16 (48)	67 (32)	
Present - High	11 (13)	24 (26)	6 (18)	41 (19)	

*n.a.* not applicable; *EDSS* Expanded Disability Status Scale; *SD* Standard Deviation; *MS* Multiple Sclerosis; *Y/N* Yes/No

*Pearson Chi-square test to categorical variables or ANOVA/Kruskal-Wallis test to continuous variables

^a^For two patients information about EDSS level was unavailable and their data are included only in total analysis

^b^Complete/incomplete

^c^Among those that reported to be currently working (EDSS 0–3, N = 51; EDSS 4–6.5, N = 17; EDSS 7–9, N = 0; Total, N = 70)

### Health-related quality of life and impact of fatigue

Descriptive data regarding health-related quality of life, assessed by the EQ-5D questionnaire and the impact of fatigue on daily living, assessed by the MFIS-BR, are reported in Table 1.

**Fig. 1:**
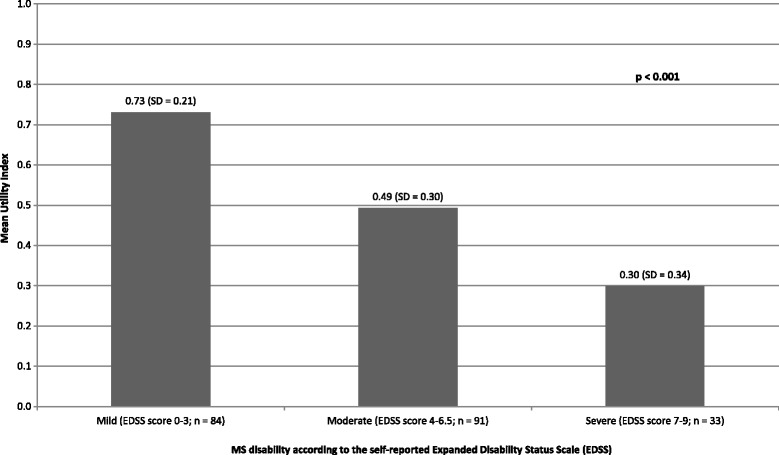
Utility by MS desability level

**Fig. 2:**
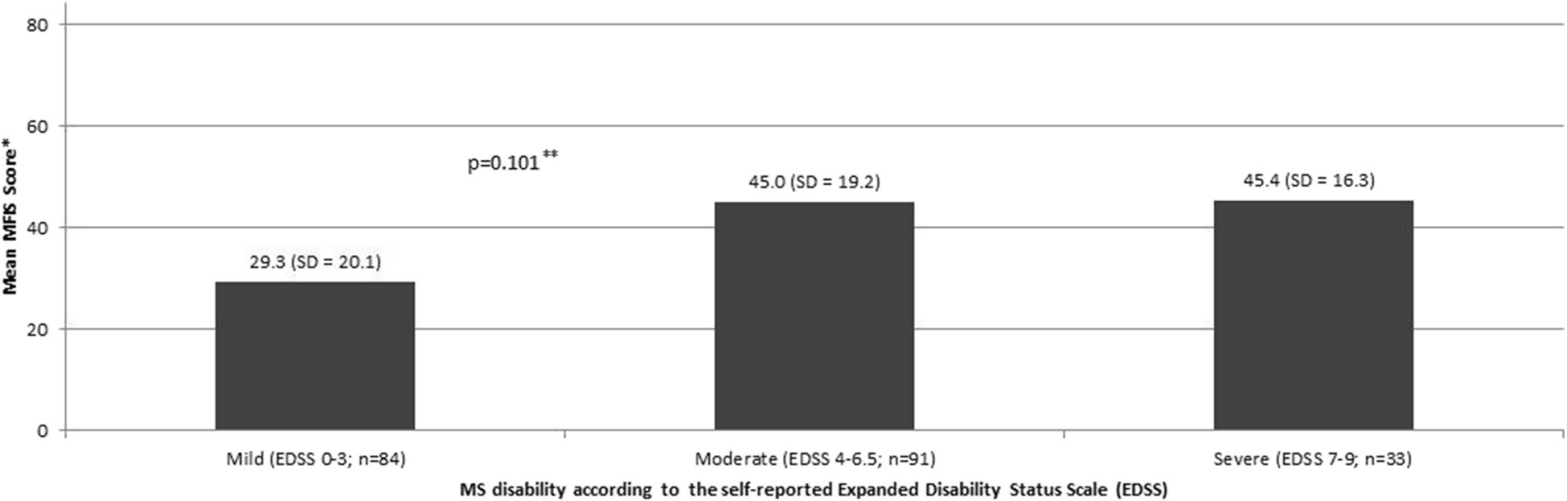
Mean MFIS total score by MS disability level

Most of the patients have shown at least some limitations in all domains evaluated by EQ-5D, except for self-care, for which 65 % of patients reported having no problems. The frequency of serious problems was higher among patients with more severe disease for all of the assessed domains, except for anxiety/depression (Table 1). When assessing self-reported quality of life through visual analogue scale (where values closer to 100 represent better state of health), the average EQ-VAS score was 71.6 (SD = 18.9) and higher scores were observed for patients with less severe disease (*p* < 0.001) (Table 1). Utility score decreased as disability

level increased (*p* < 0.001), as represented in Fig. 1. The impact of fatigue was considered absent, low and high in 49, 32 and 19 % of patients, respectively, showing that 51 % of the total sample perceived some degree of adverse impact of fatigue on daily living activities. A statistically significant difference in the distribution by category was observed in accordance to EDSS level (*p* < 0.001; Table 1). Any impact (both low and high summed) was reported by 33, 63, and 66 % of patients with mild, moderate and severe disability, respectively (Table 1). However, the same pattern was not observed among analyzed groups when MFIS was described as a mean score stratified by EDSS level (Fig. 2).

The mean MFIS total score for mild, moderate and severe patients was 29.3, 45.0, and 45.4 (38.6 in the total sample). The mean impact scores for each domain in the total sample were 20.0 (physical, range 0–36), 14.7 (cognitive, range 0–40), and 3.9 (psychosocial, range 0–8), meaning that fatigue has a proportionally higher impact in the physical than the cognitive or psychosocial domains (Fig. 2).

### Productivity loss

On working status, 34 % of patients reported being currently employed. Sick leave was required by 37 % of those who were currently working, with an annual mean duration of 141.7 days (SD = 198.7). Furthermore, 37 % of the sample had retired earlier due to MS, after a mean of 3.9 years from diagnosis (SD = 4.1). Among those, the average age of retirement was 39 years old (SD = 9.5).

Employment rate was 31, 19 and 0 % in the mild, moderate and severe disability groups, respectively, demonstrating a statistically significant difference (*p* < 0.001). The frequency of early retirement due to MS was also increased by disability severity (mild = 11 %, moderate = 53 %, and severe = 64 %; *p* < 0.001). Time from diagnosis to retirement and age of retirement were not markedly different across severity groups as shown in Table 1.

### Resource utilization

Among all patients, 26 % required hospitalization, with a mean duration of 9.02 days (SD = 9.09), and 23 % had visited an emergency department at least once in previous 12 months. Regarding outpatient visits, 98 % attended a neurologist appointment, 36 % a physical therapist, 22 % a nurse, 15 % a psychologist, 11 % a general practitioner, 3 % an occupational therapist, and 36 % reported appointments with other medical specialists in previous 12 months. When asked about tests, 63 % were submitted to magnetic resonance imaging, 12 % to lumbar puncture and 6 % to computerized tomography.

Ninety eight percent of the total sample had been prescribed disease modifying therapies and 23 % had used over-the-counter drugs. In terms of prescribed co-medications, 42 % used antidepressants, 16 % used pain relief medications, 16 % used drugs for spasticity, 11 % used drugs for insomnia, 10 % used steroids because of a recurrence, 9 % used drugs for urologic disorders, 8 % used anti-fatigue medications and immunotherapy and 2 % used medications related to cognition issues.

Considering utensils bought and other investments done, 17 % reported home modifications, 17 % acquired a walking stick, 12 % a manual wheelchair, 5 % reported car modifications and 1 % bought an electric wheelchair. Data regarding resource utilization, stratified among EDSS levels, are described in Table 2.

**Table 2:** Resource utilization per patient per year in each EDSS level

	Mild (EDSS 0–3)		Moderate (EDSS 4–6.5)		Severe (EDSS 7–9)		TOTAL	
	(N = 84)		(N = 91)		(N = 33)		(N = 210)^a^	
	Users (%)	Mean (SD)	Users (%)	Mean (SD)	Users (%)	Mean (SD)	Users (%)	Mean (SD)
Inpatient care (days)	23 %	7.53 (8.57)	33 %	10.41 (10.02)	15 %	7.40 (5.41)	26 %	9.02 (9.09)
Emergency service (visits)	20 %	4.00 (1.22)	29 %	4.77 (3.88)	15 %	2.80 (0.55)	23 %	4.29 (2.94)
Outpatient visits								
General Practitioner	8 %	3.14 (1.57)	13 %	2.83 (1.03)	15 %	2.40 (0.89)	11 %	2.83 (1.17)
Neurologist	96 %	5.09 (3.19)	100 %	5.19 (3.43)	97 %	3.75 (1.74)	98 %	4.94 (3.18)
Other specialist	32 %	7.26 (10.94)	36 %	4.12 (3.94)	45 %	15.47 (27.05)	36 %	7.52 (14.32)
Nurse	20 %	10.35 (13.40)	25 %	8.43 (6.32)	18 %	4.67 (1.63)	22 %	8.65 (9.32)
Physiotherapist	11 %	43.56 (19.33)	38 %	71.26 (45.64)	64 %	74.76 (46.40)	31 %	68.55 (43.94)
Psychologist	14 %	16.67 (19.97)	19 %	13.76 (17.87)	9 %	28.00 (45.03)	15 %	16.19 (21.31)
Occupational therapist	2 %	29.00 (26.87)	1 %	48.00 (n.a.)	9 %	42.67 (33.31)	3 %	39.00 (25.54)
Complementary tests								
MRI	60 %	1.55 (1.00)	68 %	1.56 (1.35)	55 %	1.17 (0.51)	63 %	1.50 (1.13)
CT	7 %	1.83 (1.17)	5 %	1.40 (0.89)	3 %	1.00 (n.a.)	6 %	1.58 (1.00)
Lumbar puncture	15 %	1.15 (0.38)	11 %	1.30 (0.67)	6 %	1.00 (0.00)	12 %	1.20 (0.50)
Treatment with DMTs^b^	89 %	n.a.	93 %	n.a.	61 %	n.a.	87 %	n.a.
Prescribed co-medication (days)								
Depression	32 %	355.56 (23.09)	46 %	324.29 (89.89)	55 %	360.00 (0.00)	42 %	341.79 (64.79)
Immunotherapy	1 %	12.00 (n.a.)	7 %	214.00 (162.85)	27 %	170.67 (114.75)	8 %	177.00 (135.09)
Anti-spasticity medication	5 %	280.00 (160.00)	15 %	360.00 (0.00)	48 %	349.50 (42.00)	16 %	345.65 (61.20)
Urologic	2 %	280.00 (138.56)	8 %	285.71 (127.39)	30 %	264.40 (157.25)	9 %	274.20 (137.71)
Pain	11 %	282.22 (154.34)	24 %	295.82 (124.29)	9 %	360.00 (0.00)	16 %	297.88 (126.59)
Steroids	13 %	28.36 (26.43)	10 %	32.44 (34.14)	3 %	8.00 (n.a.)	10 %	29.14 (29.04)
Insomnia	10 %	360.00 (0.00)	11 %	325.20 (110.05)	15 %	360.00 (0.00)	11 %	332.17 (94.39)
Fatigue	2 %	214.00 (206.47)	12 %	345.45 (48.24)	9 %	360.00 (0.00)	8 %	331.75 (80.87)
Cognition	0 %	n.a.	3 %	320.00 (69.28)	3 %	360.00 (n.a.)	2 %	330.00 (60.00)
Others	25 %	296.00 (122.74)	43 %	329.95 (91.30)	30 %	360.00 (0.00)	33 %	324.06 (96.97)
OTC medication	20 %	n.a.	29 %	n.a.	15 %	n.a.	23 %	n.a.
Utensils and/or modifications								
Home modifications	4 %	n.a.	19 %	n.a.	45 %	n.a.	17 %	n.a.
Car modifications	4 %	n.a.	3 %	n.a.	15 %	n.a.	5 %	n.a.
Walking stick	1 %	n.a.	35 %	n.a.	6 %	n.a.	17 %	n.a.
Wheelchair (manual)	0 %	n.a.	9 %	n.a.	52 %	n.a.	12 %	n.a.
Wheelchair (electric)	0 %	n.a.	1 %	n.a.	6 %	n.a.	1 %	n.a.
Professional care	13 %	n.a.	27 %	n.a.	33 %	n.a.	23 %	n.a.

*n.a.* not applicable; *DMTs* Disease modifying therapies; *MRI* Magnetic resonance imaging; *CT* computed tomography; *EDSS* Expanded Disability Status Scale; *OTC* Over the counter; *SD* Standard Deviation

^a^Two patients who presented no information for EDSS level are represented only on total. All analyses were made considering the total sample or total sample in each group

^b^DMTs: Interferon beta-1a, Interferon beta-1b, Glatiramer acetate and Natalizumab

### Costs

Average total direct costs per year were USD 19,012.32 (SD = 10,465.96) per patient. No statistically significant difference was observed according to MS disability level (*p* = 0.398), as described in Table 3.

**Table 3:** Mean annual cost (USD) of multiple sclerosis according to disability level

	Mild (EDSS 0–3)	Moderate (EDSS 4–6.5)	Severe (EDSS 7–9)	Total^a^	*P* value*
	(n = 84)	(n = 91)	(n = 33)	(n = 210)	
A - Resources funded by the health system, except drugs for treating MS					
Inpatient care					
Mean	42.44	82.74	27.95	57.82	0.025
SD	127.05	185.01	82.40	150.78	
Emergency service					
Mean	4.98	8.39	2.61	6.04	0.297
SD	11.95	28.49	6.71	20.45	
Consultations (visits to physicians and other healthcare professionals)					
Mean	211.84	530.43	1038.29	478.08	0.001
SD	423.80	897.53	1225.55	853.99	
Complementary exams					
Mean	126.96	145.63	86.10	128.69	0.019
SD	149.03	179.08	96.32	156.41	
Adjuvant drugs					
Mean	84.56	173.98	2527.71	506.48	0.040
SD	194.46	368.22	12,174.82	4851.34	
Total A					
Mean	**470.80**	**941.17**	**3682.67**	**1177.10**	<0.001
SD	**578.59**	**1166.34**	**12,327.31**	**5020.98**	
B - Drugs for treating Multiple Sclerosis – DMTs^b^					
Mean	**17,283.59**	**17,409.30**	**10,545.01**	**16,257.60**	<0.001
SD	**8118.31**	**6760.00**	**8853.87**	**7982.50**	
C - Resources funded by the patient					
Non-prescribed drugs (OTC medication)					
Mean	45.84	75.04	93,29	65.51	0.754
SD	136.62	226.18	303,62	209.56	
Investment and equipment					
Mean	416.71	451.84	2949.64	826.23	0.912
SD	2087.26	2148.73	7343.50	3580.73	
Professional caregiver					
Mean	258.14	493.48	2274.97	685.88	0.036
SD	947.76	1190.69	5279.98	2395.80	
Total C					
Mean	**720.68**	**1020.37**	**5317.90**	**1577.62**	0.010
SD	**2339.49**	**2520.58**	**9670.85**	**4678.00**	
Total cost					
Mean	**18,475.08**	**19,370.84**	**19,545.57**	**19,012.32**	0.398
SD	**8256.02**	**7509.87**	**19,445.75**	**10,465.96**	

*MS* Multiple Sclerosis; *DMTs* Disease modifying therapies; *EDSS* Expanded Disability Status Scale; *OTC* over the counter; *SD* standard deviation

*Significant at 0.05 Kruskal-Wallis/ANOVA tests; Values in bold represent the total cost of each category (A, B and C)

^a^Two patients who presented no information for EDSS level are represented only on total. All analyses were made considering the total sample or total sample in each group

^b^DMTs: Interferon beta-1a, Interferon beta-1b, Glatiramer acetate and Natalizumab

Costs for each MS disability level were also stratified in three different categories: A, direct medical costs funded by the health system, excluding DMTs; B, DMTs funded by the health system; and, C, direct medical and non-medical costs funded by patients (Table 3). DMTs represented around 90 % of total costs for mild and moderate MS patients. For severe patients, DMTs represented 54 % of total costs, followed by direct medical and non-medical costs funded by patients (27 %) and direct medical costs (except DMTs) funded by the health system (19 %).

In the multivariate analysis, ANOVA with partial sum of squares was employed and only EDSS level was associated with higher non-DMTs direct costs (*p* = 0.012). Other variables in the model were gender (*p* = 0.210), education (*p* = 0.134), impact of fatigue (*p* = 0.83), episode of recurrence (*p* = 0.075), having any comorbidity (*p* = 0.713), MS type (*p* = 0.227) and current activity (*p* = 0.591), which were not associated with non-DMTs direct costs.

## Discussion

The primary aim of this study was to assess the economic impact of MS from the Brazilian household and healthcare system perspectives. Secondary objectives were to assess the impact of fatigue on daily living and HRQL of MS patients.

The study was designed to generate Brazilian data about MS impact; however, it enrolled patients from South and Southeastern regions, which have potential differences from other regions in terms of socio-demographic characteristics and access to specialized MS healthcare services. Despite these differences, prevalence reported for the Southeast region is higher than those reported for the Northeast and Midwest regions [5], making data representative from the vast majority of Brazilian MS patients. Moreover, the study also demonstrated the impact of MS on health-related quality of life and the impact of fatigue on daily living, providing a broader analysis of the economic and psychosocial impact of MS on patients and their families than other previously published Brazilian studies that addressed particular aspects of the disease [19–23].

Findings indicated an average total direct cost per year of USD 19,012.53 (SD = 10,466.07) per patient. In several countries, previous studies have demonstrated that MS costs are substantially high [3]. Depending on the perspective taken and the severity of the disease, MS costs ranged from USD 3162 to USD 77,938 per patient per year [45]. However, cost comparison studies are not recommended due to differences in the methodological approach, such as data collection, type of resources included, assessment of resources, patients included, sampling process and quality of the analysis [3, 45]. In addition, cost drivers can vary across geographies probably due to the significant differences in the availability of services and use of resources across countries [45]. For example, differences in DMTs costs reflect differences in utilization across countries, in study populations included in analyses, and in period of data collection (where earlier studies have low DMTs costs because they were conducted prior to the widespread use of disease modifying drugs) [3].

In the present study, DMTs accounted for the majority of direct expenditures, especially among patients with lower levels of disability, representing around 90 % of total costs for mild and moderate MS patients and 54 % of total costs for severe MS patients. It was also observed that expenses with medical (except DMTs) and non-medical resources are higher among patients with more severe disease.

These findings corroborate results from studies conducted worldwide which have shown that severe disability is associated with increased costs of hospitalizations, consultations, laboratory tests and other drugs, although the cost of immunomodulatory drugs is low [8, 9]. This fact would explain why no statistically significant difference was observed when the total cost was stratified by MS disability level. The analysis of productivity loss has shown that sick leave and early retirement due to MS were common among the studied population and they increased according to the progression of disability. In addition, the employment rate was significantly low (34 % of the total sample with 100 % of those with severe disability being unemployed or retired), particularly considering a mean age of 40.7 years (SD = 11.5) and a national unemployment rate of 5.6 % in the general Brazilian population (according to official national data) [46]. Similar results were found by other authors [35, 47]. Furthermore, the majority of patients in all EDSS levels had been in school for at least 10 years, a similar result to the one found by Fragoso et al. [48] where patients have studied for an average of 12.4 years, evidencing that despite the high schooling levels, a high frequency of unemployment or early retirement is observed among MS Brazilian patients.

It was also possible to observe through the health-related quality of life analysis, expressed as utility measures, that worsening in disability level led to lower utility scores – representing a worse assessment by patients of their own health state. The same results were previously described by other authors [3, 8, 9, 14–18, 35]. Although these findings corroborate others previously described, it is important to note that utility index was calculated using an algorithm developed for the United Kingdom population since an algorithm developed for the Brazilian population was not available at the time of data analysis [49].

Some degree of adverse impact of fatigue on daily activities was perceived by 51 % of the sample, particularly related to the physical domain. In Brazil, Nogueira et al. [11] found higher frequencies of self-reported impact of fatigue, about 69 %, using MFIS-BR, and Mendes et al. [12] also found higher frequencies, 67.4 %, although he used the Fatigue Severity Scale. Other previous studies also have shown the association between level of disability and impact of fatigue, considering different study designs and sample sizes [3, 50, 51].

Finally, it is important to note that this study protocol was planned based on methods used on the TRIBUNE study, conducted on five European countries [35] and Canada [14]. In all countries where a similar MS study was conducted, including Brazil, most of the enrolled patients were female, as expected for a disease that affects at least twice as many more women than men [3].

Considering severity level (EDSS), only the United Kingdom results were similar, with the majority of patients classified between 4 and 6.5 [35]. In other countries, considering the combined results for Europe, most patients were classified in EDSS levels between 0 and 3 [35]. Brazilian data also showed a greater frequency of severe disability (EDSS 7–9) when compared to other countries, even with a small number of patients included in this category.

In relation to the economic impact of MS, study data corroborated those observed in other countries, showing that increased healthcare-related costs is directly proportional to increased disability level (if DMT drugs are considered separately). When only costs with DMTs were considered, results for all countries are also similar, showing that costs become inversely proportional to increased disability level [14, 35]. Data on quality of life also corroborated results from Europe and Canada, where patients with the most severe disease showed worst quality of life [14, 35].

## Conclusion

This study can make an important contribution to the existing MS literature in Brazil once it provided a broader analysis of the economic and psychosocial impacts of MS on patients, their families and on the Brazilian health system. Findings highlighted the considerable economic impact of the disease, both in terms of disease modifying therapies and other MS management costs. When patients move upwards on the disease severity scale, costs with health resources other than DMTs are significantly increased. Worsening disability also had an important influence on quality of life and self-perceived impact of fatigue on daily living. Further studies are necessary to assess not only direct but also indirect costs of MS in Brazil.
